# Is helicopter transferal in the “drip-and-ship” approach for endovascular treatment the better choice? A retrospective analysis of transfer times

**DOI:** 10.3389/fneur.2025.1582098

**Published:** 2025-07-01

**Authors:** Evgeniia Lagno, Johanna Ernst, Andreas Flemming, Frauke Raab, Friedrich Götz, Vesta Brauckmann, Christian Macke, Hans Worthmann

**Affiliations:** ^1^Department of Neurology, Hannover Medical School, Hannover, Germany; ^2^Interdisciplinary Emergency and Disaster Medicine, Hannover Medical School, Hannover, Germany; ^3^Department of Anesthesiology and Surgical Intensive Care Medicine, KRH Hospital Siloah, Hannover, Germany; ^4^Institute of Diagnostic and Interventional Neuroradiology, Hannover Medical School, Hannover, Germany; ^5^Department of Trauma Surgery, Hannover Medical School, Hannover, Germany

**Keywords:** acute ischemic stroke, endovascular treatment, inter-hospital transfer, emergency rescue helicopter, ground ambulance, telestroke network

## Abstract

**Background:**

For patients with large vessel occlusion (LVO) admitted to primary stroke centers (PSC) without neuro-interventional capabilities, timely transfer to comprehensive stroke centers (CSC) is crucial. In this study, we compared the transport time of ground- and air-based transfer for patients receiving endovascular treatment at our CSC.

**Methods:**

In a retrospective cohort study, consecutive ischemic stroke patients with LVO who were transferred ground- or air-based to our CSC between October 2018 and December 2022 were examined. 170 patients with LVO from five PSCs within a radius of 55 to 85 km to the CSC were included. Patients were transported either with an emergency rescue helicopter (ERH), a ground ambulance (GA), GA accompanied by an emergency physician vehicle (EPV), or in a mobile intensive care unit (MICU) and were accordingly divided into air-based (61 patients) and ground-based (109 patients) main transport groups.

**Results:**

The analysis revealed a significant difference between air- and ground-based transport groups (75 vs. 82 min, *p* = 0.01). After calculating the transport time in relation to the covered ground distance, air-based transport was shorter by a median of 0.15 min per kilometer. In a comparison of the individual means, ERH was faster than GA and EPV (both *p* < 0.001). Only few transports were done by MICU and they mainly showed very long transfer times. The complication rates were generally low with only minor complications and no deaths reported in both groups. However, they were more frequently observed in the land-based transport group (20.2% vs. 8.2%, *p* = 0.04).

**Conclusion:**

In the present analysis, air-based transport was faster than ground-based transport for the secondary transfer of patients with stroke due to LVO in the observed regional conditions. Both air- and land-based transport appear to be safe. No serious complications occurred during transport, while complications were more frequent in the ground-based transport group.

## 1 Introduction

Among neurological diseases, stroke has the greatest emergence of disability-adjusted life years. It is one of the leading causes of death worldwide (1). In Germany, approximately 270.000 people suffer a stroke each year, of which 85% are of ischemic cause (2).

In 2015, seminal studies demonstrated that endovascular treatment (EVT) in a narrow time window of up to 6 h after symptom onset (3) is a highly effective therapy for acute ischemic stroke due to large vessel occlusion (LVO). Since expansion of the time window for EVT up to 24 h in 2018, the number of patients who receive EVT and benefit from the procedure has largely increased (4, 5). In primary stroke centers (PSC) without 24/7 EVT-availability, whenever an EVT would be considered, rapid secondary patient transport to a comprehensive stroke center (CSC) should be initiated. For the best outcomes of stroke patients, a well-organized transport chain starting at the PSC up to the CSC is crucial. This could be achieved by close collaboration between these hospitals, as well as different emergency medical services, which include both air- and ground-based transport options.

Air-based transport has the potential of reducing transport time because it overcomes the restrictions of ground-based transport means such as infrastructure limitations or rush hours. However there is still insufficient evaluation, especially in settings with moderate distances between PSC and CSC when both air- and ground-transport means are available. Also, to date, there is no clear criteria for selecting the means of transport for transferring stroke patients. Naturally those criteria would differ between regions due to differences in the available infrastructure. Timely air-based transport is often hampered by differences in transport-protocols between different regions (6), especially since the time needed to prepare an air-based transport is dependent on the available infrastructure and is usually longer than that for ground-based transport vehicles (7). There are also differences in the available ground transport choices which may considerably affect the transport time. To make the comparison more difficult, patients’ characteristics and other regional and hospital-specific factors may also affect the choice of the transport vehicle. In fact, our analysis might facilitate the development of a standardized regional workflow for transferring stroke patients in comparable regional settings which would make the transport decision of stroke patients more straightforward thus generally enhancing acute stroke care by making it much more efficient. In order to achieve that, in this study, we compare the transport duration for different means of inter-hospital transfer to our CSC to achieve EVT for patients with ischemic stroke due to LVO. In addition, we evaluated the complications reported during transfer in the different transport groups as well as possible effects of the transport time on clinical and procedural EVT-outcomes. Since various regional and hospital-specific factors have a significant influence on the choice of transport mode, this study conducted an analysis at a single center to achieve a high consistency and similarity of the observed conditions.

## 2 Methods

### 2.1 Study population

**Figure 1:**
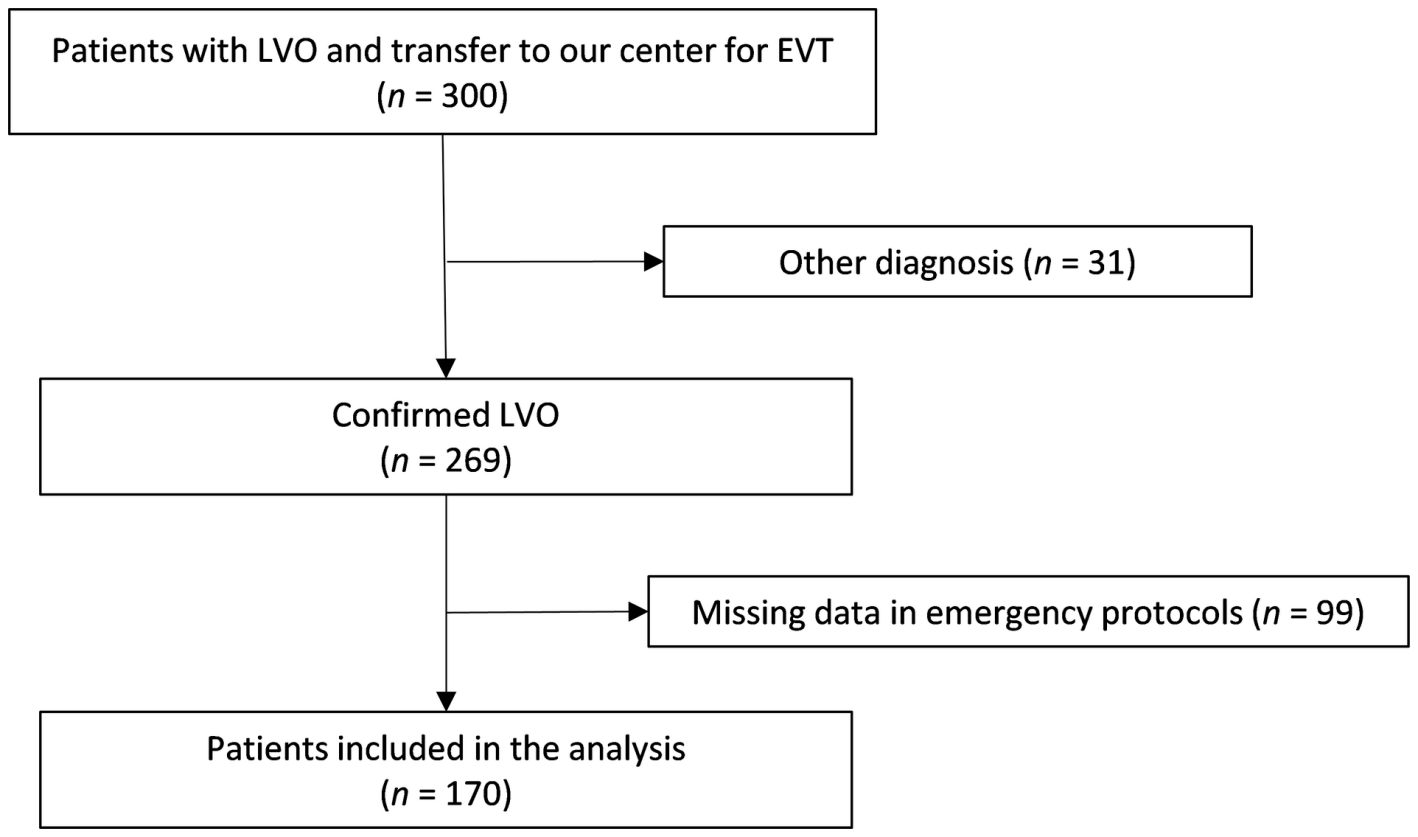
Study flow diagram. LVO, large vessel occlusion; EVT, endovascular treatment.

**Figure 2:**
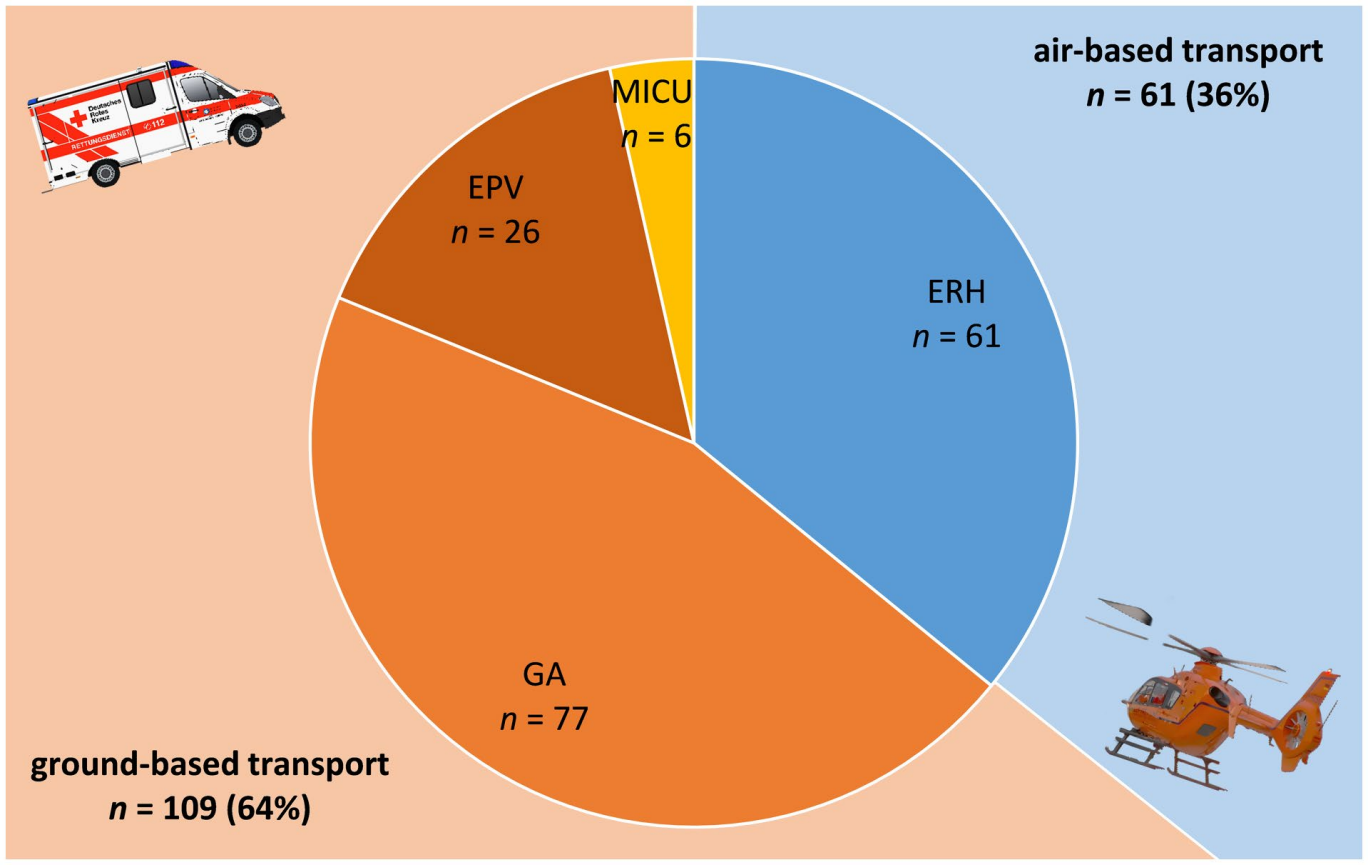
Graphical representation of the different transport groups. ERH, emergency rescue helicopter; GA, ground ambulance; EPV, emergency physician vehicle; MICU, mobile intensive care unit. The number of patients is included for each transport vehicle. The percentages of the two main groups (ground- and air-based) are presented in parenthesis.

We included patients with ischemic strokes due to LVO who were secondarily transferred to our CSC at Hannover Medical School for EVT between October 1st, 2018 and December 31st, 2022. Patients were included from hospitals that regularly cooperated with our CSC for secondary transfers for EVT. Transfers within the urban area and/or hospitals that do not provide air transport due to short distances within the city and district of Hannover were not analyzed. Therefore, patients transported from five PSCs within a 56.4–84.1 km ground-distance radius to our center were included in the study. The decision which transport mean to choose was at the discretion of the PSC. However, consultation with our CSC usually took place beforehand over the telestroke network or directly by phone and the different means of transportation were discussed. Out of 300 transferred patients, LVO was confirmed in 269 patients. Of these, 170 had full hospital records in regard to emergency reports and thus were included in the analysis (Figure 1). The mean air-distance was 50.7 ± 6.4 km. Patients were transported either by an emergency rescue helicopter (ERH; *n* = 61 patients), a ground ambulance (GA; *n* = 77 patients), GA accompanied by an emergency physician vehicle (EPV: *n* = 26 patients) or a mobile intensive care unit (MICU; *n* = 6 patients) and were thus divided into two main groups; air-based transport (61 patients, 36%) vs. ground-based transport (109 patients, 64%) (Figure 2). The contributions of each PSC to these groups as well as their distances to the CSC are listed in Table 1. Of note, two of the PSCs (PSC 2 and PSC 5) had no neurological departments and three of them (PSC 1, PSC 3 and PSC 4) had a regional stroke unit but without EVT capability. It is also worth noticing that no patients were transported air-based from PSC 4.

**Table 1:** Air- and ground-distances of the primary stroke centers (PSC) from the CSC and their contribution (presented as percentages) to the overall study population as well as both transport groups.

PSC	Distance		Patients		
	Air	Ground	All, *n* (%)	Air, *n* (%)	Ground, *n* (%)
PSC 1	49.1 km	58.5 km	95 (55.9)	38 (40)	57 (60)
PSC 2	65.6 km	84.1 km	19 (11.2)	10 (52.6)	9 (47.4)
PSC 3	57.2 km	77.7 km	19 (11.2)	10 (52.6)	9 (47.4)
PSC 4	44.3 km	60.2 km	28 (16.5)	0 (0)	28 (100)
PSC 5	43 km	56.4 km	9 (5.3)	3 (33.3)	6 (66.7)

All methods were carried out in accordance with relevant guidelines and regulations. The conduction of the study was approved by the Ethics Committee of Hannover Medical School (Ethics Committee of Hannover Medical School, Hannover, Germany, Approval No. 10102_BO_K_2021). In this retrospective observational analysis, informed formal consent was waived after weighing up interests in accordance with the Lower Saxony Rescue Service Act (NRettDG) and the Lower Saxony Data Protection Act (NDSG).

Patients’ demographics and clinical characteristics including medical history, imaging and EVT results assessed using the Thrombolysis In Cerebral Infarction (TICI) score, modified Rankin Scale (mRS) pre-stroke and at discharge, National Institutes of Health Stroke Scale (NIHSS) pre-transfer and at discharge, as well as type of transport vehicle and key time-based metrics such as transport order time, door-out-time, arrival time and time of initiation of EVT were collected. In addition, data regarding complications during transport and air- and ground- distances from the presenting PSC to the CSC were examined in the study.

The patients’ transport ordering time, the patients’ arrival time at the CSC and the time of initiation of EVT (groin-time) were recorded. The patients’ transport-time (TT) was calculated as the difference between the transport ordering time at the PSC and the patient’s arrival time at the CSC. TT was compared between the transport types. The TT is dependent on the distance to be traveled and inherits a bias against ground-based transport vehicles having to navigate through the available road network until they reach their destination, which might significantly increase their travel distance. Therefore, we defined the corrected transport time (cTT) as TT divided by the ground distance between the PSC and the CSC and compared it between the groups. Additionally, we studied the observed complications during transport as well as the influence of TT on the patients’ radiological and clinical outcome; in particular, the success of EVT assessed using the TICI score and the mRS and NIHSS scale on discharge.

Since multiple factors may have affected the choice of the transport type in each PSC, we further investigated the subgroup of patients transported from PSC 1 in a multivariate analysis. This hospital contributed the highest patients’ number to the overall population and had both air- and ground-based transport options available at the site (40% vs. 60%, respectively).

### 2.2 Statistical analysis

Statistical analysis was done using IBM SPSS Statistics Version 29.0.1.0 (IBM Corporation, Armonk, NY, United States). Numbers and percentages were used to describe categorical variables and median and 25th to 75th percentile for non-normally and mean ± standard deviation (SD) for normally distributed continuous variables. Boxplots with Tukey whiskers were generated unless reported otherwise. Group comparisons were done using the Mann–Whitney-U-Test and Kruskal-Wallis-Test for non-normally and two-tailed t-test for normally distributed continuous data and the Chi-square test for comparison of categorical data. A *p* value of < 0.05 was considered statistically significant. The effect of TT on patients’ outcomes was studied using logistic regression models. A linear regression model for the subgroup of patients from PSC 1 was established including transport type as a dependent variable and TT and potential confounding factors as independent variables.

## 3 Results

### 3.1 Overall characteristics

Overall, clinical characteristics of the patients are summarized in Table 2. The median TT was 78 min. No relevant differences between air- and ground-based transported patients were observed regarding sex, age, comorbidities, stroke severity, need for repeat imaging, location of cerebral occlusion and EVT rate. Furthermore, no differences in the prevalence of unknown time window or the rate of endotracheal intubations between both groups were detectable. Also no differences between the initial NIHSS and NIHSS at destination with a median difference between the two of 0 points were found. TT, cTT and the rate of observed complications during transport differed in regard to the mode of transport. Since we had to exclude a large amount of patients due to missing data in the emergency protocols we compared the group of included and excluded patients and importantly, found no differences in the patient characteristics between the two groups. To conclude, no significant effect on the results of our study are expected due to the exclusion of patients. The results of this comparison can be found in the Supplementary material S1.

**Table 2:** Baseline patients’ characteristics.

Baseline characteristics		All (*n* = 170)	Air-based transport (*n* = 61)	Ground-based transport (*n* = 109)	*p* value
Female, *n* (%)		86 (50.6)	29 (47.5)	57 (52.3)	0.552
Age, years (25th-75th pct.)		77 (66.5–83)	78 (62–82)	77 (67.8–84)	0.294
Hypertension, *n* (%)		147 (86.5)	50 (82.0)	97 (89.0)	0.199
Atrial fibrillation, *n* (%)		75 (44.1)	24 (39.3)	51 (46.6)	0.348
Diabetes mellitus, *n* (%)		39 (22.9)	12 (19.7)	27 (24.8)	0.448
History of stroke, *n* (%)		28 (16.5)	7 (11.5)	21 (19.3)	0.189
Hyperholesterolemia, *n* (%)		82 (48.2)	26 (42.6)	56 (51.4)	0.273
Coronary artery disease, *n* (%)		29 (17.1)	11 (18)	18 (16.5)	0.801
NIHSS initial, mean ± SD		14 ± 6	15 ± 6	14 ± 6	0.321
NIHSS at destination, mean ± SD		16 ± 8	16 ± 8	15 ± 8	0.506
Pre-mRS, *n* (%)	0	108 (63.5)	42 (68.9)	66 (60.6)	0.411
	1	19 (11.2)	4 (6.6)	15 (13.8)	
	2	30 (17.6)	9 (14.8)	21 (19.3)	
	3	8 (4.7)	3 (4.9)	5 (4.6)	
	4	5 (2.9)	3 (4.9)	2 (1.8)	
Unknown time window, *n* (%)		44 (25.9)	16 (26.2)	28 (25.7)	0.938
Endotracheal intubation, *n* (%)		14 (8.2)	7 (11.5)	7 (6.4)	0.25
TT, minutes (25th-75th pct.)		78 (69.5–90)	75 (68–84)	82 (70.8–95)	**0.01**
cTT, min/km (25th-75th pct.)		1.27 (1.11–1.42)	1.18 (1.02–1.31)	1.33 (1.18–1.46)	**<0.001**
Re-imaging at destination, *n* (%)		54 (31.8)	16 (26.2)	38 (34.9)	0.246
Cerebral occlusion, *n* (%)	ICA	14 (8.2)	5 (8.2)	9 (8.3)	0.488
	MCA	87 (51.2)	27 (44.3)	60 (55.0)	
	ACA	0 (0.0)	0 (0.0)	0 (0.0)	
	VA	2 (1.2)	0 (0.0)	2 (1.8)	
	BA	11 (6.5)	5 (8.2)	6 (5.5)	
	PCA	1 (0.6)	0 (0.0)	1 (0.9)	
	ICA + MCA	55 (32.4)	24 (39.3)	31 (28.4)	
Thrombectomy, *n* (%)		143 (84.1)	51 (83.6)	92 (84.4)	0.892
Complications, *n* (%)		27 (15.9)	5 (8.2)	22 (20.2)	**0.04**

Data are presented as median with 25th to 75th percentiles non-normally distributed continuous variables and as mean ± SD for normally distributed continuous variables and percentage for categorical variables. NIHSS, National Institutes of Health Stroke Scale. Pre-mRS, Modified Rankin Scale; primarily documented at the presenting hospital; ICA, internal carotid artery; MCA, middle cerebral artery; ACA, anterior cerebral artery; VA, vertebral artery; BA, basilar artery; PCA, posterior cerebral artery; TT, transport time; cTT, corrected transport time; pct, percentile; SD, standard deviation. *p* < 0.05 was considered significant regarding the differences between air- and ground-transported patients and bold p-values represent values reaching this statistical significance.

### 3.2 Transport time and corrected transport time

**Figure 3:**
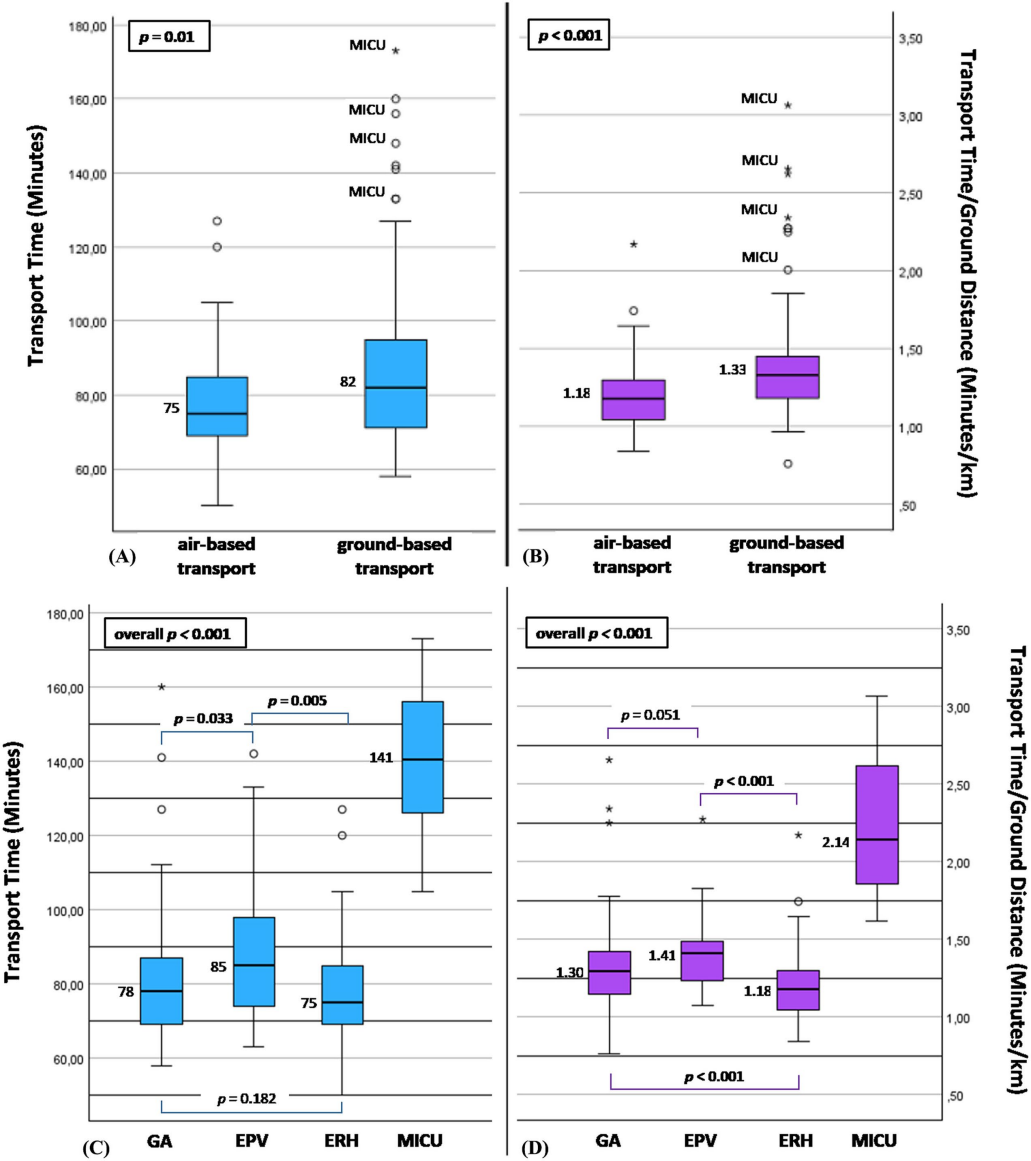
**(A,B)** Air-based vs. ground-based transport groups: **(A)** transport time measured in minutes; **(B)** corrected transport time measured in minutes/km; of note most of the extreme values occurred for MICU. **(C,D)** Subdivision according to individual transport types: **(C)** transport time measured in minutes; **(D)** corrected transport time measured in minutes/km. ERH, emergency rescue helicopter; GA, ground ambulance; EPV, emergency physician vehicle; MICU, mobile intensive care unit.

The median TT was 75 min in the air-based transport group vs. 82 min in the ground-based transport group (*p* = 0.01; Figure 3A). The median cTT was 1.18 min/km in the air-based transport group vs. 1.33 min/km in the ground-based transport group (*p* < 0.001; Figure 3B). Figures 3C,D presented TT and cTT within the individual transport groups (GA, EPV, ERH and MICU), respectively. A comparison of cTT between those groups showed a significant difference between GA and ERH (cTT for GA 1.3 min/km vs. ERH 1.18 min/km (*p* < 0.001)). Of notice, the MICU group showed very long transport times for both TT (105–173 min) and cTT (1.62–3.07 min/km). However, due to the very low number of patients transported using MICU, it had only little influence on the results of the overall ground-based transport group, explaining most of its extreme values (Figures 3A,B). A figure showing the results of the comparison of the two main transport groups excluding MICU can be found in the Supplementary material S2.

### 3.3 Association between transport time and outcomes

We used logistic regression models to study any possible association between TT / cTT and patients’ outcomes. The study showed no association of TT/ cTT with the success of EVT assessed using the TICI – scale (TICI 1 - 2a vs. 2b - 3), nor it showed any significant association between TT or cTT and the NIHSS (NIHSS 0–5 vs. > 5) or mRS (mRS 0–3 vs. 4–6) scores assessed at the time of hospital discharge.

### 3.4 Transport complications

Complications were more frequently observed in the ground-based transport group compared to the air-based group (ground-based vs. air-based: 20.2% vs. 8.2%, *p* = 0.04). Comparing the individual groups, complications were most frequently reported in the EPV cases (EPV 34% vs. ERH 8.2%, GA 15.6%, MICU 16.7%). However, the overall rate of complications was low and no serious or fatal complications or endotracheal intubations have occurred during transport. Furthermore, no association could be found between the reported complications and the administration of i.v. thrombolysis (IVT) prior to transport (*p =* 0.645 and 0.244 for air-based and ground-based transport, respectively). Complications are summarized in Table 3.

**Table 3:** List of complications during transport.

Complications	Overall *n* = 27	ERH *n* = 5	GA *n* = 12	EPV *n* = 9	MICU *n* = 1
High blood pressure	8.2%	4.9%	10.4%	7.7%	16.7%
Low blood pressure	4.1%	3.3%	2.6%	11.5%	0.0%
Agitation	0.6%	0.0%	0.0%	3.8%	0.0%
Nausea and vomiting	4.1%	4.9%	1.3%	11.5%	0.0%
Hyperglycemia	0.6%	0.0%	1.3%	0.0%	0.0%
Afib with rapid ventricular response	1.8%	0.0%	3.9%	0.0%	0.0%
Others	1.8%	3.3%	0.0%	3.8%	0.0%

Data is represented in percentages. Afib, atrial fibrillation. ERH, emergency rescue helicopter; GA, ground ambulance; EPV, emergency physician vehicle; MICU, mobile intensive care unit.

### 3.5 Subgroup analysis of PSC 1

**Figure 4:**
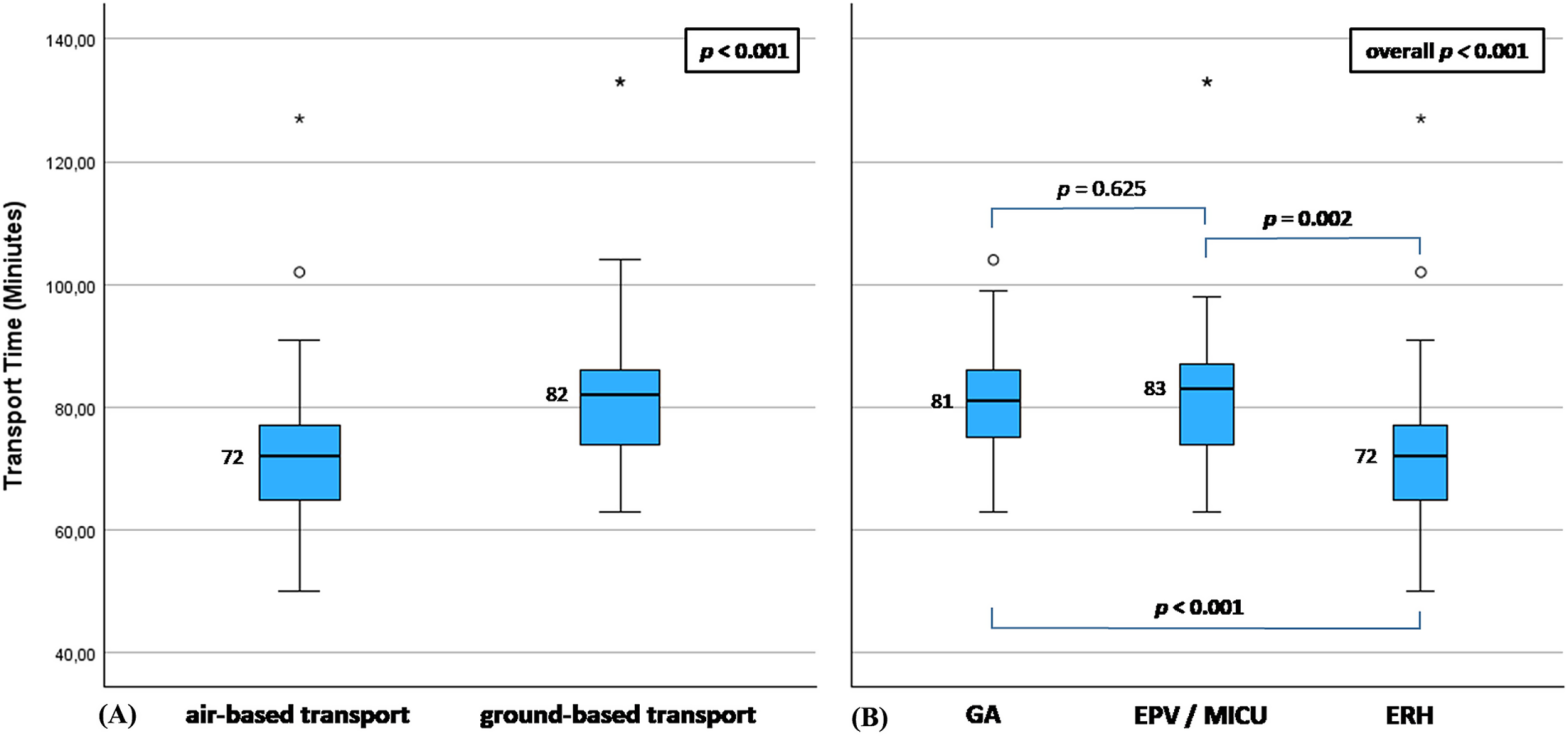
Boxplot representation of the analysis results of the subgroup of hospital 1. **(A)** air-based vs. ground-based transport groups. **(B)** Subdivision into three main transport types. ERH: emergency rescue helicopter; GA, ground ambulance; EPV, emergency physician vehicle; MICU, mobile intensive care unit. *p* < 0.05 was considered statistically significant.

To account for the individual processes of each of the included PSCs and to avoid heterogeneity, PSC 1 containing the majority (95 patients) of transferred cases was investigated in a subgroup analysis. The observed difference in the TT between air-based and ground-based transport groups remained statistically significant (TT for air-based vs. ground-bound transport: 72 vs. 82 min, *p* < 0.001; Figure 4A). In the comparison between the different transport means, a significant difference between ERH and both of GA and EPV and no difference between GA and EPV/MICU was found (Figure 4A), similar to cTT in the overall group. Of note, only one patient was transported from PSC 1 using MICU and was included in the EPV group in this analysis. The baseline characteristics of the patients in this subgroup did not differ, while they can be found in the Supplementary material S3.

**Figure 5:**
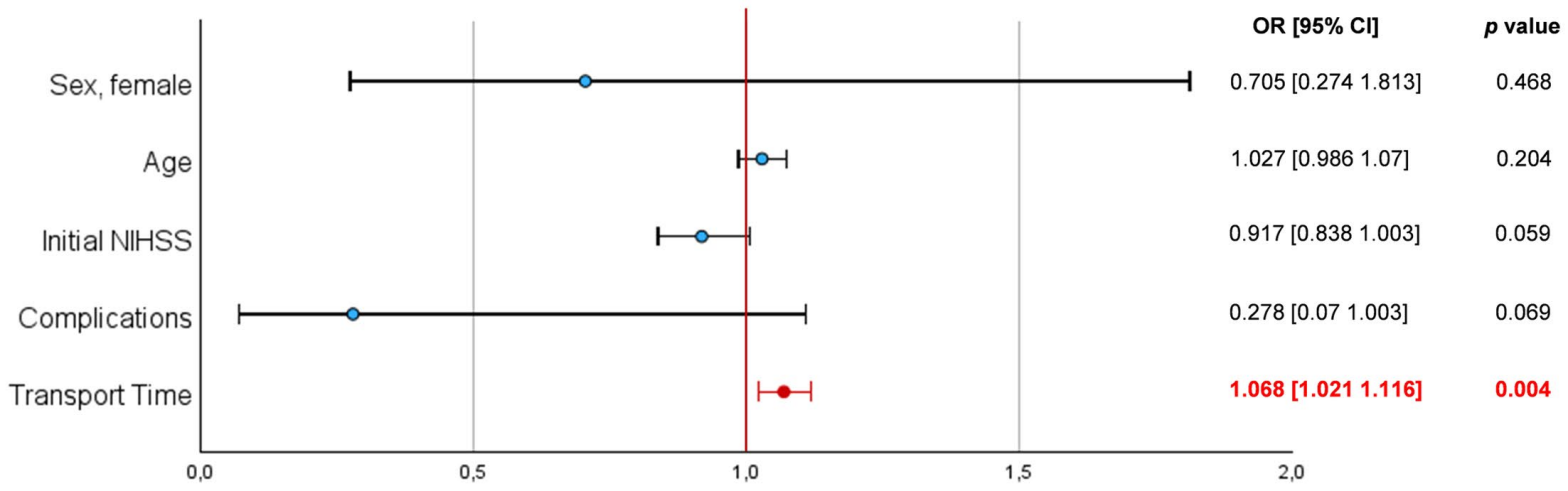
Multivariate regression analysis of the hospital 1 subgroup with Forest-plot representation of the calculated odds ratios (OR) and 95% confidence intervals (CI).

To investigate the influence of possible confounding factors in this subgroup, we performed a multivariate linear regression analysis, in which only TT was found to be associated with the transport type (*p* = 0.004) with an odds ratio (OR) of 1.068 and 95% confidence interval (CI) of [1.021 1.116] (Figure 5).

## 4 Discussion

Efficient time processes for patients with acute ischemic stroke due to LVO represent a critical healthcare priority and have a significant impact of time on EVT outcomes (8, 9). Evaluating and refining the transfer process of patients prior admitted to centers without EVT capabilities is crucial (8, 10), as it can positively affect transfer times and subsequently patient outcomes. In our study, we aimed to investigate the secondary patient transfers according to a drip-and-ship transfer model in a regional setting with longstanding cooperation between the PSCs and the CSC. Regional studies are best suited to do this kind of investigations since differences in regional infrastructure as well as transport-protocols naturally influence the efficiency of the different transport means despite their potential availability. Based on our regional setting of transfers with medium distances between the PSCs and our CSC, we carried out an analysis of available data, which could serve as an example for regions with similar settings. We observed that air-based transport by ERH was significantly faster than ground-based transport. The overall difference in median TT was 7 min. This is much less than most of the calculated transfer time reduction when helicopters were used to transfer patients with stroke between the PSC and the CSC in 2019 in the Danish drip-and-ship model reported by Behrndtz et al. (11), in which those time savings differed according to the geographical location in Denmark. The transfer time reduction ranged from 4 min for the Capital area to 44 min in the southern region. These differences might be at least partially explained by the differences in the organizational structure of the healthcare and emergency systems between the studied regions. On the other hand, in a previous study (12), Kunte et al. reported that in 205 patients’ transfers for ischemic stroke treatment between August 2014 and September 2019 in Nashville, TN, USA, inter-hospital transfers by air were predicted to be faster than by ground only for distances over 40 miles. Accordingly, Paoli et al. (13) suggested not to use helicopter transfers for distances less than 50 km in a study including 115 helicopter air ambulance operations for patients with various diseases in 2018 in the Padua region in Italy. In both studies, logistics surrounding air-based transport played an important role and explained the lack of time advantage of air-based transport for short travel distances. This also represents the explanation that in our studied region, no use of secondary air-based transport is generally reported for hospitals located closer to our center than the studied PSCs. To account for the distance between the PSCs and CSC, in our study we analyzed a corrected version of transport time by correcting it to the ground distance needed for an ambulance to travel in order to reach our center and found the median cTT to be 1.18 min/km and 1.33 min/km for air-based and ground-based transport, respectively. This resulted in a time saving median difference of 0.15 min for air-based transport for each kilometer of possible ground travel distance. Ground-based transport using MICU, however, did not follow this rule and showed very long transport times. This is probably due to the inherited complexity of its initiation and due to it being a specialized transport form for very sick patients who cannot be transported otherwise. This kind of transport vehicles are scarce and usually require a special transport order and longer distances until reaching the PSC. Also, in our case, it is stationed at our CSC and has thus to travel the ground distance to the PSC twice. No correlation with day-time was witnessed in the limited number of MICU- cases. As MICU had very long transfer-times, we would preferably avoid it unless there is a clear advantage of it as a decision on a case-by-case basis. Although ERH might also have a longer setup time, this process is much more standardized than MICU. The higher travel speed of ERH negates much of the time needed for its setup resulting in short overall transfer times, which are comparable, or, as in our study, even faster than the almost always readily available GA vehicles.

Another aspect of air-based transport is the availability and the higher costs to the healthcare system when routinely using ERH for patients’ transport. This has been analyzed by Coughlan et al. (14) who concluded that in England, air-based transfer of rural patients with a confirmed ischemic stroke becomes cost-effective when travel time is reduced by at least 60 min compared with ground-based transport. Although this threshold might not be transferable to the German health system, the use of the corrected TT as a mean for predicting the time benefit of air-based transport might make such economical calculations more straightforward.

In our analysis, we could not find any association between TT and cTT and patient’s procedural outcomes or clinical status at the time of discharge. This could be due to the relatively short travel distances (12). With longer transport times and larger distances, however, a notable effect on outcomes should be expected (7, 15).

Although all of the PSCs in our study shared the lack of capabilities for EVT, they differed in terms of neurological organizational structure. Some of them had a dedicated stroke unit with an onsite neurology consultant, while others were supported by a telestroke network from our CSC. Telestroke networks play a significant role in facilitating swift and precise evaluations according to the stroke unit principles of patients initially presenting to remote clinics, which is essential for fast decision making and includes a transfer to an EVT- capable center when needed (16, 17). However, the heterogeneous structures of the PSCs in our study might have substantially influenced the patients’ transfer process. We performed a subgroup analysis after selecting the PSC with the most transfers as well as an optimized organization of processes. Here, a multivariate regression model was performed identifying TT to be the only factor that differed between air-based and ground-based transport groups. With an odds ratio of 1.068 (CI 1.021–1.116), however, this difference in favor of the air-based transport was rather small which might be explained by the relatively short ground distance of this PSC to our center (Table 1). Nevertheless, having a positive effect of air-based transport at such distance stands in contrast to a previous study by Gangadharan et al. (7). In this study, employed on consecutive patients transferred to a comprehensive stroke center (CSC) for endovascular clot retrieval in Australia, the distance at which the extra speed of an aircraft made up for the delays involved in its preparation was found to be 299 km. However, transport time metrics were examined in rural and sparsely populated areas with minimal reported transfer distance of 167 km and limited access to healthcare facilities, which does not reflect the easily accessible healthcare infrastructure in northern Germany. In those circumstances, many external factors such as availability and location of endovascular clot retrieval or weather conditions would have a much greater impact alongside patient-specific factors in shaping the transfer order decisions. In our cohort, none of the 170 patients required endotracheal intubation during transport. This could be explained by shorter distances in our study compared to Australia (7) when in some cases intubation had to be performed leading to prolonged transport times. Additionally, we found no correlation between TT and patient outcomes at discharge (mRS, NIHSS), nor between TT and EVT results as assessed with the TICI- scale. In a study on a large cohort of 615 patients, Pallesen et al. (18) found that IVT, higher heart rate and lower oxygen saturation at departure were associated with the amount of medical interventions during transport. In that study, approximately 11.1% of patients deteriorated during transfer of which around 60% had to be intubated during transport. The overall event rate of complications during transport in our study was 15.9%. However, we observed no major complications and no instances of cardiopulmonary resuscitation, emergency endotracheal intubation or death. Various mild complications were noted with higher occurrence during ground-based transport, particularly using EPVs. We found no significant difference between patients who received IVT and those who did not. Medical interventions during transfer were overall low.

As an alternative to the studied secondary transfer of patients from PSC to CSC, there are also other approaches for reducing TT such as the “flying/driving interventionalists” model where EVT is achieved through the deployment of a CSC neurointerventionalist performing the EVT at the PSC after driving or flying. This model was previously shown to be feasible in the rural regions of southwest Bavaria in Germany in the setting of the regional telestroke network (19). Interestingly, there were no differences in terms of technical success or complication rates. On the other hand, it requires very close cooperation between the PSC and CSC, as there is a high level of organizational effort in terms of personnel and infrastructure (16, 19).

## 5 Limitations

The results described in our paper apply specifically to the studied population and great care is needed when extrapolating them to other regions and/or healthcare systems, especially since many different regional and hospital-specific factors may affect the choice of transport means. Some of these factors relate to differences in healthcare infrastructure, which varies very much between regions and/or countries. However, the analysis of a single region, as in the present study, is particularly meaningful due to the similarity of the conditions observed. Although the transport time for MICU was much longer than that for other means of transport, no definitive conclusions can be drawn about MICU, as this type of transport occurred in only six cases. Also, a proportion of patients had to be excluded from our study due to lack of prehospital data. However, we do not expect this to substantially change the study results, as the data are clear and consistent, just as in the subgroup analysis for PSC. Additionally, it is worth noticing, that although the corrected transport time seems to be a more accurate way of measuring transport time, larger studies including patients from different regional settings are needed in order to validate it as a generally accepted transport time metric and whether the results also hold for distances outside of the range included in our study. Finally, a significant part of the analyzed patients’ transports in our study occurred during the COVID-19 pandemic, which might have affected the availability of transport means as well as the actual transport times, since possibly identification of COVID-19 positive patients might have been required prior to transport in some cases. However, although significant changes with prioritizing COVID transfers over others has been described in Italy (20), no significant changes were largely observed in Germany (21) and the impact on stroke severity overall was small (22).

## 6 Conclusion

Our analysis reveals that air-based transport is faster than ground-based transport in the secondary transfer of patients with acute ischemic stroke due to LVO in the observed regional conditions. The introduced corrected transport time (cTT) might be more accurate in describing the differences between types of transport. We found no association between transport times (TT) and EVT outcomes. In our study population, no serious complications occurred during transport while minor complications were more frequently observed in the ground-based transport group.

## Data Availability

The raw data supporting the conclusions of this article will be made available by the authors, without undue reservation.

## Glossary

ACA: Anterior cerebral artery
BA: Basilar artery
CSC: Comprehensive stroke center
cTT: Corrected transport time
EPV: Emergency physician vehicle
ERH: Emergency rescue helicopter
EVT: Endovascular treatment
GA: Ground ambulance
ICA: Internal carotid artery
LVO: Large vessel occlusion
MCA: Middle cerebral artery
MICU: Mobile intensive care unit
mRS: Modified Rankin scale
NIHSS: National institutes of health stroke scale
PCA: Posterior cerebral artery
PSC: Primary stroke center
SD: Standard deviation
TICI: Thrombolysis in cerebral infarction score
TT: Transport time
VA: Vertebral artery
