# Supplementary material for: Is helicopter transferal in the “drip-and-ship” approach for endovascular treatment the better choice? A retrospective analysis of transfer times
Source: Front Neurol. 2025 Jul 1;16:1582098. doi: 10.3389/fneur.2025.1582098 (PMC12260230; doi:10.3389/fneur.2025.1582098)
Supplement: Supplementary file 1 [file Data_Sheet_1.doc]

| **Baseline characteristics** | **all**  **(*n* = 269)** | **included  (*n* = 170)** | **excluded (*n* = 99)** | ***p* value** |
| --- | --- | --- | --- | --- |
|
| **Female. n (%)** | 127 (47.2) | 86 (50.6) | 41 (41.4) | 0.146 |
| **Age. years (25th-75th pct)** | 77 (65-83) | 77 (66.5-83) | 76 (63-83) | 0.569 |
| **NIHSS initial. mean ± SD** | 14 ± 6 | 14 ± 6 | 13 ± 6 | 0.351 |
| **Unknown time window. n (%)** | 68 (25.3) | 44 (25.9) | 24 (24.2) | 0.884 |
| **Endotracheal intubation for transport. n (%)** | 21 (8.1) | 14 (8.2) | 7 (8.0) | 0.938 |

Supplement 1. Comparison of the main characteristics of patients included in our study and those who had to be excluded from it due to incomplete data. Statistical analysis was performed using Pearson chi-squared test for categorical variables (presented as percentages) and Mann–Whitney-U-Test for non-normally distributed continuous variables (presented as median with 25th to 75th percentiles) and two-tailed t-test for normally distributed continuous variables (presented as mean ± SD). NIHSS: National Institutes of Health Stroke Scale. p < 0.05 was considered significant
