# Supplementary material for: Is helicopter transferal in the “drip-and-ship” approach for endovascular treatment the better choice? A retrospective analysis of transfer times
Source: Front Neurol. 2025 Jul 1;16:1582098. doi: 10.3389/fneur.2025.1582098 (PMC12260230; doi:10.3389/fneur.2025.1582098)
Supplement: Supplementary file 2 [file Data_Sheet_2.doc]

Supplement 1. Boxplot representation of the transport times analysis results of air-based vs. ground-based transport groups excluding MICU. On the left transport time measured in minutes; on the right corrected transport time measured in minutes/km; the comparison was calculated using Mann–Whitney-U-Test. MICU: mobile intensive care unit. p < 0.05 was considered statistically significant.
