# Supplementary material for: Is helicopter transferal in the “drip-and-ship” approach for endovascular treatment the better choice? A retrospective analysis of transfer times
Source: Front Neurol. 2025 Jul 1;16:1582098. doi: 10.3389/fneur.2025.1582098 (PMC12260230; doi:10.3389/fneur.2025.1582098)
Supplement: Supplementary file 3 [file Data_Sheet_3.doc]

| **Characteristic** | | | **all**  **(*n* = 95)** | **air-based transport  (*n* = 38)** | **ground-based transport  (*n* = 57)** | ***p* value** |
| --- | --- | --- | --- | --- | --- | --- |
|
| **Female. n (%)** | | | 49 (51.6) | 16 (42.1) | 33 (57.9) | 0.131 |
| **Age. years (25th-75th pct)** | | | 80 (68-84) | 78 (61.8-82.3) | 80 (69-85) | 0.19 |
| **Hypertension. n (%)** | | | 80 (84.2) | 9 (23.7) | 6(10.5) | 0.085 |
| **Atrial fibrillation. n (%)** | | | 38 (40) | 24 (63.2) | 33 (57.9) | 0.608 |
| **Diabetes mellitus. n (%)** | | | 27 (28.4) | 29 (76.3) | 39 (68.4) | 0.403 |
| **History of stroke. n (%)** | | | 19 (20) | 5 (13.2) | 14 (24.6) | 0.173 |
| **Hyperholesterolemia. n (%)** | | | 48 (50.5) | 18 (47.4) | 30 (52.6) | 0.615 |
| **Coronary artery disease. n (%)** | | | 16 (16.8) | 6 (15.8) | 10 (17.5) | 0.823 |
| **NIHSS initial. mean ± SD** | | | 15 ± 6 | 16 ± 5 | 14 ± 6 | 0.137 |
| **NIHSS at destination center. mean ± SD** | | | 16 ± 7 | 16 ± 7 | 16 ± 7 | 0.702 |
| **Pre-mRS. n (%)** | | 0 | 53 (55.8) | 22 (57.9) | 31 (54.4) | 0.501 |
|  | | 1 | 13 (13.7) | 3 (7.9) | 10 (17.5) |
|  | | 2 | 19 (20) | 7 (18.4) | 12 (21.1) |
|  | | 3 | 5 (5.3) | 3 (7.9) | 2 (3.5) |
|  | | 4 | 5 (5.3) | 3 (7.9) | 2 (3.5) |
| **Unknown time window. n (%)** | | | 17 (17.9) | 5 (13.2) | 10 (17.5) | 0.566 |
| **Endotracheal intubation for transport. n (%)** | | | 5 (5.3) | 3 (7.9) | 2 (3.5) | 0.348 |
| **Transport time. minutes (25th-75th pct)** | | | 77 (69-85) | 72 (64.8-77.5) | 82 (74-86.5) | **< 0.001** |
| **Re-imaging at destination center. n (%)** | | | 26 (27.4) | 8 (21.1) | 18 (31.6) | 0.26 |
| **Cerebral occlusion. n (%)** | | ICA | 11 (11.6) | 3 (7.9) | 8 (14) | 0.358 |
|  | | MCA | 49 (51.6) | 17 (44.7) | 32 (56.1) |
|  | | ACA | 0 (0) | 0 (0) | 0 (0) |
|  | | VA | 0 (0) | 0 (0) | 0 (0) |
|  | | BA | 6 (6.3) | 3 (7.9) | 3 (5.3) |
|  | | PCA | 0 (0) | 0 (0) | 0 (0) |
|  | ICA+MCA | | 29 (30.5) | 15 (39.5) | 14 (24.6) |  |
| **Thrombectomy. n (%)** | | | 83 (87.4) | 32 (84.2) | 51 (89.5) | 0.449 |
| **Complication during transport. n (%)** | | | 17 (17.9) | 3 (7.9) | 14 (24.6) | **0.038** |

Supplement 3. Baseline patient characteristics of the patients transported from Primary stroke center 1 (PSC1). Statistical analysis was performed using Pearson chi-squared test for categorical variables (presented as percentages) and Mann–Whitney-U-Test for non-normally distributed continuous variables (presented as median with 25th to 75th percentiles) and two-tailed t-test for normally distributed continuous variables (presented as mean ± SD). NIHSS: National Institutes of Health Stroke Scale. Pre-mRS: Modified Rankin Scale; primarily documented at the presenting hospital. ICA: internal carotid artery; MCA: middle cerebral artery; ACA: anterior cerebral artery; VA: vertebral artery; BA: basilar artery; PCA: posterior cerebral artery. p < 0.05 was considered significant regarding the differences between air- and ground-transported patients and bold p-values represent values reaching this statistical significance.
